# Effectiveness of blended learning to improve medical students’ communication skills: a randomized, controlled trial

**DOI:** 10.1186/s12909-025-06938-w

**Published:** 2025-03-14

**Authors:** Sebastian Gross, Kurt Wunderlich, Armon Arpagaus, Christoph Becker, Flavio Gössi, Benjamin Bissmann, Samuel K. Zumbrunn, Michael Wilde, Sabina Hunziker

**Affiliations:** 1 Medical Communication and Psychosomatic Medicine, University Hospital Basel, Basel, Switzerland; 2 Faculty of Medicine, University of Basel, Klingelbergstrasse 23, Basel, CH-4031 Switzerland; 3 Medical Polyclinic, University Hospital Basel, Basel, Switzerland

**Keywords:** Online learning, E-learning, Blended learning, Communication

## Abstract

**Background:**

This study aimed to evaluate whether a blended learning course improves medical students’ communication skills compared to a face-to-face only lecture.

**Methods:**

After completing a face-to-face lecture on communication skills, 2nd year medical students were gender-matched and randomized to either an intervention receiving an interactive video-based online learning module covering the content of the previous lecture and including a knowledge assessment or a control group only receiving a knowledge assessment. The primary endpoint was students’ knowledge about communication techniques assessed by a predefined score from 0 to 100. Secondary outcomes included students’ feedback and satisfaction on a scale from 0 to 5. Additionally, qualitative analysis of free-text responses to patient case vignettes was conducted.

**Results:**

One hundred sixty-four medical students were included in the final analysis (64% female). The intervention group had significantly higher knowledge (mean, SD points) (73.6 ± 10.7 versus 56.7 ± 15.3, adjusted difference 17.02, 95%CI 12.95 to 21.1, *p* < 0.001) and reported higher satisfaction (4.3 ± 0.9 versus 3.5 ± 1.0, difference 0.78, 95%CI 0.48 to 1.07, *p* < 0.001) compared to the control group. Qualitative analysis of free-text responses also revealed improvements in patient-centered communication techniques in the intervention group.

**Conclusions:**

Blended learning significantly enhances medical students’ communication skills and satisfaction compared to traditional lecture-based learning and may thereby contribute to the development of future knowledge and practices to improve patient-centered care.

**Clinical trial number:**

Not applicable.

**Supplementary Information:**

The online version contains supplementary material available at 10.1186/s12909-025-06938-w.

## Background

Effective communication is vital in providing patient-centered care, as it reduces patients’ psychological distress, enhances satisfaction, improves compliance and impacts recovery and hospital readmission rates [1–3]. Conversely, the majority of medical errors originates from communication failure including poor communication with patients [4–6]. The substantial influence of communication suggests that teaching of communication skills should be integral to medical curricula [7]. Teaching these skills is challenging due to the need to balance technical competence with empathetic engagement. Methods include observation- based professional feedback (real time or recorded with video-assisted debriefing), self-assessment using structured tools, role-playing scenarios, and role modeling by observing experienced professionals in practice [8, 9]. In recent years, particularly since the onset of the emerging COVID pandemic, medical education increasingly adopted blended learning formats, combining face-to-face and virtual or online learning [2, 10, 11]. This approach offers advantages such as efficiency, overcoming time and distance barriers, accessibility to a greater number of students, cost-effectiveness and it may also be preferred by medical students [2, 9, 12–14]. Recent systematic reviews support the effectiveness of blended learning for teaching clinical skills to medical students and health professionals [2, 15]. However, these reviews emphasize the challenge of the evaluation process of blended learning, which is often based on self-assessment questionnaires and thus subject to bias representing an important knowledge gap [2, 11, 15, 16]. Importantly, to proof causality there is need for randomized, controlled trials to evaluate the impact of blended learning on objectively measurable outcomes, such as the understanding and application of communication techniques in medical education.

At the University of Basel, the longitudinal curriculum “Social and Communicative Competencies” provides medical students with the opportunity to receive systematic and evidence-based training in physician-patient communication from their first year of study through to graduation. This curriculum is integrated into the formal medical education program and also an integral part of formal exams for medical students in Switzerland [17]. As part of the curriculum the course “Basics of Medical Communication” is conducted during the 2nd bachelor’s year. It traditionally comprises six face-to-face lectures of two hours each, distributed across the semester rather than delivered consecutively, to maximize engagement and learning retention. Prior to the introduction of the intervention, this course did not include a knowledge assessment, although communication competencies were assessed as part of a standardized examination at the end of the 3rd bachelor’s year.

This study aimed to evaluate whether a blended learning format could enhance 2nd year medical students’ understanding and application of communication techniques and increase their satisfaction compared to the existing course. A randomized, controlled design including a knowledge assessment was used to address the lack of evidence. For this purpose, a new, interactive video-based online learning module was introduced.

## Methods

### Study design and randomization

This randomized, controlled trial evaluated the effect of a blended learning course on medical students’ communication skills. Participation was voluntary and supplementary to the face-to-face lecture. Students were gender-matched and randomized 1:1 to either an intervention or a control group after a lecture on medical communication techniques using a computer-generated random number list via REDCap^®^. Gender-matched randomization ensures balanced gender distribution but may lead to minimal group size differences [18]. The intervention group completed an interactive video-based online learning module including a knowledge assessment, while the control group only completed the knowledge assessment.

The online learning intervention was designed as web-based training (WBT) that was delivered via an open-source learning management system (OpenOlat) [19]. It was accessible to students through a secure access link and password. Data were extracted anonymously with all identifying information such as names, dates of birth and email addresses irreversibly deleted to prevent data tracing. The local ethics committee (Ethics Committee of Northern and Central Switzerland) was consulted for formal clarification of responsibility and determined that ethical approval was not required (Req-2024-00639).

### Participants

All 2^nd^ year medical students (*n* = 225) at the University of Basel, Switzerland who volunteered were eligible to participate in in the trial conducted in April 2024. As the basics of medical communication are an integral part of the 2^nd^ year of the longitudinal communication curriculum at the University of Basel, this annual course provided an ideal context for introducing a new online learning module. Medical students at the University of Basel are already accustomed to using online learning tools from other courses of their curriculum.

### Study flow and description of intervention

The online learning module was developed collaboratively by the medical communication department at the University Hospital Basel and the University of Basel. The University provided a dedicated time slot in the students’ schedules for conducting the study. Students were invited to participate voluntarily via announcements made during previous lectures two weeks prior to the study supplemented by written invitations sent through the students’ internal WhatsApp chat three days before the trial. The trial was conducted in a lecture hall in person to minimize dropout rates and prevent protocol violations. Students were instructed to bring an electronic device and headphones. Upon arrival, students were randomized at the entrance and given access to either the intervention or the control group but were blinded to their group allocation. Students in the intervention group completed an interactive, video-based online learning module including a knowledge assessment, taking approximately 90 min to complete both. A completion gating feature ensured that all students would fully watch the videos. The control group received only the knowledge assessment without the explanatory videos or video-based training.

The online learning intervention covered the content of the face-to-face lecture in accordance with the 2^nd^ year curriculum, focusing on five main domains: [1] basics of general communication techniques (e.g. initiating patient conversation) [2], the concept of physician- and patient-centered communication [3], the “WEMS” communication techniques, an acronym for “Waiting”, “Echoing,” “Mirroring,” and “Summarizing” [4, 20] the “NURSE” communication techniques for addressing emotions, an acronym for “Naming,” “Understanding,” “Respecting,” “Supporting,” and “Exploring“ [8, 20, 21], structuring medical information (i.e., “book metaphor” communication technique) [22]. Each domain was addressed though a three-step process:

1. Theory acquisition via animated explanatory videos in cartoon style created with a graphic design tool (Canva [23]).
2. Practice of communication techniques using video-based training including interactive videoclips (i.e. acted-out doctor-patient conversation whose course could be influenced by mouse click).
3. The knowledge assessment was conducted through two gamified tasks: a multiple-choice quiz and a video annotation exercise. Gamification was implemented by incorporating a dynamic leaderboard, which displayed real-time rankings of participants based on their performance. The educational objective of incorporating leaderboards was to enhance students’ motivation and engagement by fostering a sense of competition and achievement, ultimately encouraging active participation [24, 25]. The leaderboard aimed to increase engagement and foster a sense of friendly competition. The quizzes were delivered using the OpenOlat software. While the primary quiz format was multiple-choice questions (MCQs), the video annotation task required participants to apply communication skills by identifying and analyzing specific behaviors in recorded patient-physician interactions. These tasks were designed to be both educational and interactive, ensuring that students could actively engage with the material rather than passively consume it. Also, we did employ additional gamification elements such as alternative question formats (e.g., open-response questions, fill-in-the-blank).

A comprehensive description of all online learning contents can be found in Appendix 1.

### Quantitative outcomes

The knowledge assessment consisted of five sections, each corresponding to one of the abovementioned knowledge domains. To normalize the scores in each section, the raw score (e.g. 1 point for every correct answer) was multiplied by 100 and divided by the maximum achievable points. This process generated a sub-score ranging from 0 to 100, with 0 indicating the lowest possible knowledge and 100 the highest. The primary outcome was the students’ overall knowledge across the 5 domains ranging from 0 to 100. A detailed description of all scoring methods is provided in Appendix 1.

Secondary outcomes included feedback on [1] students’ personal engagement with the teaching contents [2], satisfaction with the face-to-face lecture, and [3] satisfaction with the online learning module or the electronical knowledge assessment in the control group, respectively. These were rated on a 5-point Likert scale (1: Strongly Disagree; 2: Disagree; 3: Neither Agree nor Disagree; 4: Agree; 5: Strongly Agree).

### Qualitative outcomes

The knowledge assessment included two written case vignettes of patients expressing emotions [1]. a patient subtly indicating concern and [2] a patient expressing anger due to waiting times (see Table 1). Students were asked to respond to these patient statements in a free-text format. These responses were subsequently rated by two members of the research team adapted from the Verona Coding Scale, categorizing answers as either *reducing space*, *providing space using a specific patient-centered communication technique* (i.e. WEMS or NURSE), or *providing space* in another way [26]. Disagreements of ratings were resolved through continuous discussions between the raters until a consensus was found. These qualitative ratings were for descriptive purpose only and did not influence the overall knowledge score (see Appendix 1). Furthermore, all students were given the opportunity to provide written free-text feedback at the end of the course.

**Table 1:** Qualitative analysis of students’ free-text answers to two written case vignettes

Case vignette	Communication strategy	Rating of response (Table 1)	Communication technique	Example of students’ free-text answers	Student’s randomization
Concerned patient (WEMS-exercise): Physician: *“Good day*, *Mr. M. What brings you here today?”* Patient: *“Yes*, *good day*, *doctor. Well*, *I would like to have a general check-up done. Just to see whether everything is alright.”* How would you, as the physician, react here?	Pysician-centered communication	Reduce space	Giving medical information	*“Whether I would recommend the check-up depends on your age. There are certainly young patients who opt for regular check-ups and may then pay for them themselves depending on their health insurance.”*	Control
			Physical Examination	I would start with the general check-up.	Control
			Closed question	*“A general check-up is certainly never a bad idea. Are there any specific complaints that prompt you to go for a check-up?”*	Control
			Focused question	*“Alright. When was your last check-up?”*	Control
	Patient-centered communication	Provide space through WEMS	Waiting (**W**EMS)	I would wait with a questioning gaze to see if the patient expresses any possible reasons for their concerns.	Intervention
			Echoing (W**E**MS)	*“Whether everything is alright?”*	Intervention
			Mirroring (WE**M**S)	*“It seems to me that you’re concerned that something isn’t right?”*	Intervention
			Summarizing (WEM**S**)	*“So*, *you would like to have a general check-up to see if everything is okay…"*	Control
		Provide space other	Open question	*“Everything alright? Do you have a particular concern?”*	Intervention
Angry patient (NURSE-exercise): Physician: *“Good day*, *Mrs. M. I’m here for the ward round.”* Patient: *“About time! This hospital is an absolute joke!”* How would you, as the physician, react here?	Pysician-centered communication	Reduce space	Ignoring	I would ignore the remark and proceed with the ward round.	Control
			Closed question	*“Oh*, *did you have to wait long*, *Mrs. M.?”*	Control
			Focused question	*“Thank you for your patience! I’m here for you now. How is your health condition today?”*	Control
			Apology / justification	*“I’m sorry you had to wait. We’re quite busy at the moment*, *and I wasn’t able to get to you sooner.”*	Control
	Patient-centered communication	Provide space through NURSE	Naming (**N**URSE)	*“I get the impression that you’re upset?”*	Intervention
			Understanding (N**U**RSE)	*“I can imagine that you’re upset due to the long waiting times.”*	Intervention
			Respecting (NU**R**SE)	*-*	-
			Supporting (NUR**S**E)	*“I’ll see what I can do to prevent delays in the future.”*	Control
			Exploring (NURS**E**)	*“Could you tell me what you mean by that?”*	Intervention
		Provide space other	Open question	*“What happened?”*	Intervention

Abbreviations: WEMS, Waiting, Echoing, Mirroring, Summarizing; NURSE, Naming, Understanding, Respecting, Supporting, Exploring

### Statistical analysis

Regarding sample size considerations, we used a convenient sample consisting of all eligible 2^nd^ year medical students at the University of Basel, Switzerland. To characterize the study population, descriptive statistics including frequencies and percentage of categorical data, and means and standard deviations for continuous variables were used as appropriate. Baseline characteristics were compared between randomization arms. Univariate logistic and linear regression was used to compare outcomes between randomization arms. As a sensitivity analysis, multivariate regression models adjusted for age and sex were calculated to adjust for student characteristics. A *p*-value of < 0.05 (two-tailed) was considered statistically significant. STATA 15.0 (Stata Corp., College Station, TX, USA) was used for all analyses.

## Results

### Baseline characteristics

**Fig. 1:**
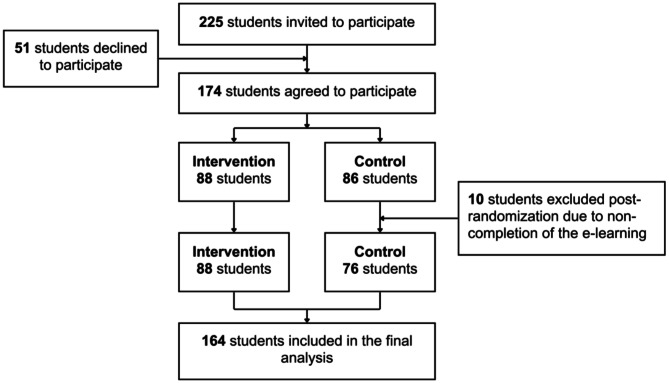
Flow chart

A total of 174 (77.3%) students agreed to participate and 164 were included in the final analysis. A detailed study flow is shown in Fig. 1. Students’ age and sex were comparable between randomization arms with a mean age of 22 years (SD ± 1.9) and 64% were female (Table 2).

**Table 2:** Association of the online learning intervention with baseline characteristics and outcomes

		Control	Intervention	OR or difference (95%CI)	*p*-value	Adjusted OR or difference (95%CI)*	*p*-value
*N*		76	88				
Baseline Characteristics							
Female sex, *n* (%)		48 (63%)	57 (65%)		0.83		
Age, mean (SD)		21.8 (1.9)	22.1 (1.8)		0.25		
Primary Outcome							
Students’ overall knowledge (from 0-100), mean (SD)		56.7 (15.3)	73.6 (10.7)	16.97 (12.94, 21.01)	**< 0.001**	17.02 (12.95, 21.1)	**< 0.001**
Secondary Outcomes							
Students’ knowledge within the domain *communication basics* (from 0-100), mean (SD)		61.4 (21.3)	80.4 (19.6)	19.06 (12.76, 25.37)	**< 0.001**	19.26 (12.93, 25.59)	**< 0.001**
Students’ knowledge within the domain *patient- vs. physician-centered communication* (from 0-100), mean (SD)		65.3 (23.1)	81.7 (14.9)	16.39 (10.47, 22.31)	**< 0.001**	16.64 (10.71, 22.56)	**< 0.001**
Students’ knowledge within the domain *WEMS communication techniques* (from 0-100), mean (SD)		52.4 (24.9)	71.1 (19.9)	18.77 (11.87, 25.67)	**< 0.001**	18.85 (11.92, 25.79)	**< 0.001**
Students’ knowledge within the domain *NURSE communication techniques’* (from 0-100), mean (SD)		50.0 (21.1)	73.5 (20.3)	23.48 (17.09, 29.88)	**< 0.001**	23.46 (17, 29.92)	**< 0.001**
Students’ knowledge within the domain *communication structure* (from 0-100), mean (SD)		54.2 (39.7)	61.4 (30.4)	7.15 (-3.68, 17.99)	0.19	6.92 (-3.85, 17.68)	0.21
Students’ responses to WEMS case vignette, *n* (%)	Reduce space	47 (62%)	27 (31%)	0.27 (0.14, 0.52)	**< 0.001**	0.27 (0.14, 0.53)	**< 0.001**
	Patient-centered technique	13 (17%)	45 (51%)	5.07 (2.45, 10.51)	**< 0.001**	4.96 (2.38, 10.33)	**< 0.001**
	Provide space	16 (21%)	16 (18%)	0.83 (0.38, 1.81)	0.64	0.88 (0.4, 1.93)	0.75
Students’ responses to NURSE case vignette, *n* (%)	Reduce space	48 (63%)	36 (41%)	0.4 (0.21, 0.76)	**0.005**	0.41 (0.22, 0.78)	**0.006**
	Patient-centered technique	7 (9%)	33 (38%)	5.91 (2.43, 14.39)	**< 0.001**	5.77 (2.36, 14.12)	**< 0.001**
	Provide space	21 (28%)	19 (22%)	0.72 (0.35, 1.47)	0.37	0.73 (0.35, 1.49)	0.38

*adjusted for age, sex. Abbreviations: OR, odds ratio; CI, confidence interval; SD, standard deviation; n, number; WEMS, Waiting, Echoing, Mirroring, Summarizing; NURSE, Naming, Understanding, Respecting, Supporting, Exploring

### Primary and secondary outcomes

#### Primary outcome: students’ overall knowledge

**Fig. 2:**
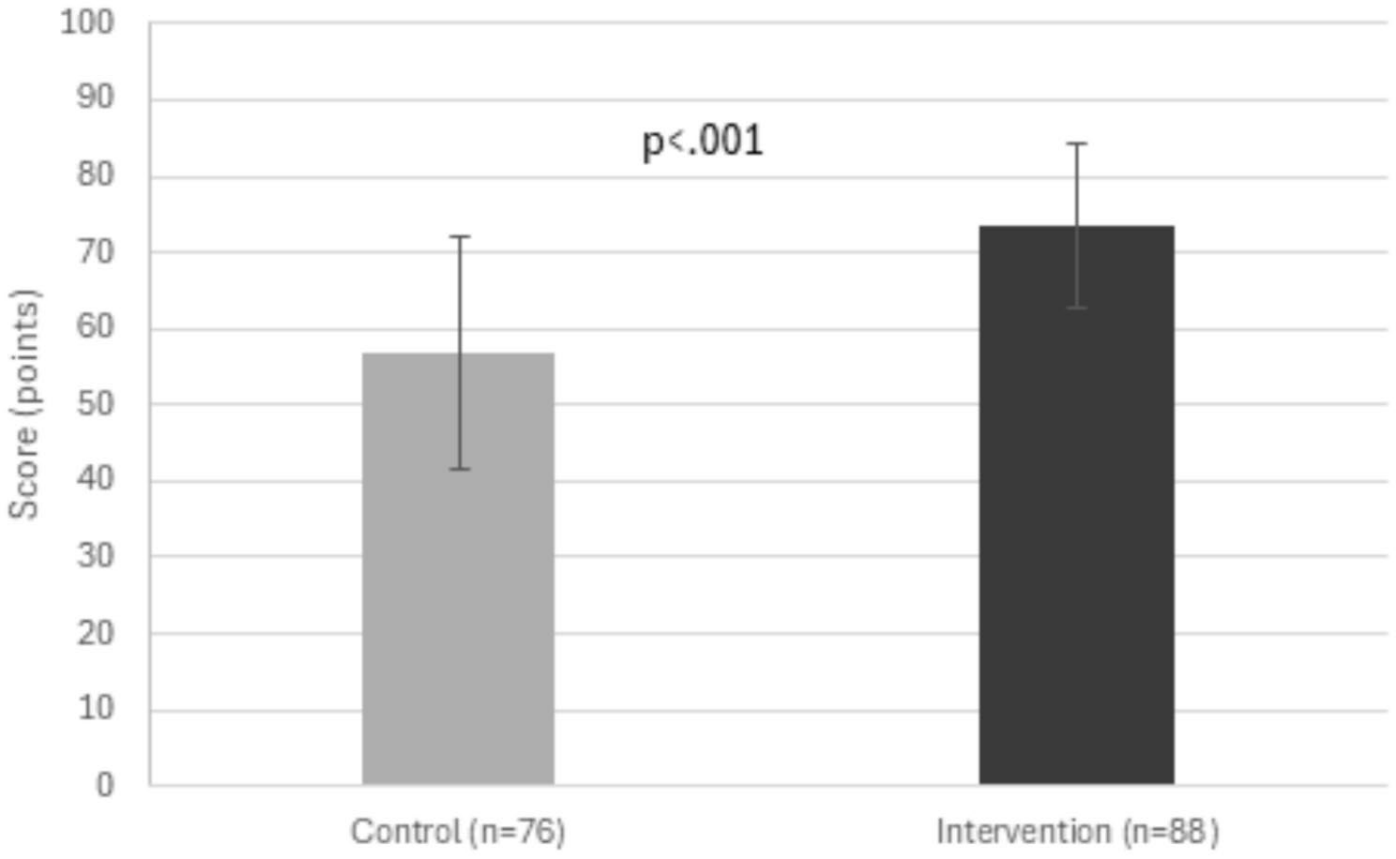
Students’ mean knowledge scores **Figure 2** shows the mean knowledge assessment scores (from 0-100) of students in the control group and intervention group, respectively

The intervention group showed significantly higher communication related knowledge compared to the control group (73.6 ± 10.7 versus 56.7 ± 15.3, adjusted difference 17.02, 95%CI 12.95 to 21.1, *p* < 0.001) (Fig. 2; Table 2).

#### Secondary outcomes: students’ knowledge within different domains

The intervention group showed significantly higher knowledge regarding the domains *basics of medical communication* (80.4 ± 19.6 versus 61.4 ± 21.3, adjusted difference 19.26, 95%CI 12.93 to 25.59, *p* < 0.001), *patient- and physician-centered communication* (81.7 ± 14.9 versus 65.3 ± 23.1, adjusted difference 16.64, 95%CI 10.71 to 22.56, *p* < 0.001), *WEMS communication techniques* (71.1 ± 19.9 versus 52.4 ± 24.9, adjusted difference 18.85, 95%CI 11.92 to 25.79, *p* < 0.001), and *NURSE communication techniques* (73.5 ± 20.3 versus 50 ± 21.1, adjusted difference 23.46, 95%CI 17 to 29.92, *p* < 0.001) compared to the control group. However, students’ knowledge regarding the domain *communication structure* did not significantly differ between groups (61.4 ± 30.4 versus 54.2 ± 39.7, adjusted difference 6.92, 95%CI -3.85 to 17.68, *p* = 0.21) (Table 2).

#### Students’ quantitative feedback on the online learning and the face-to-face lecture

**Fig. 3:**
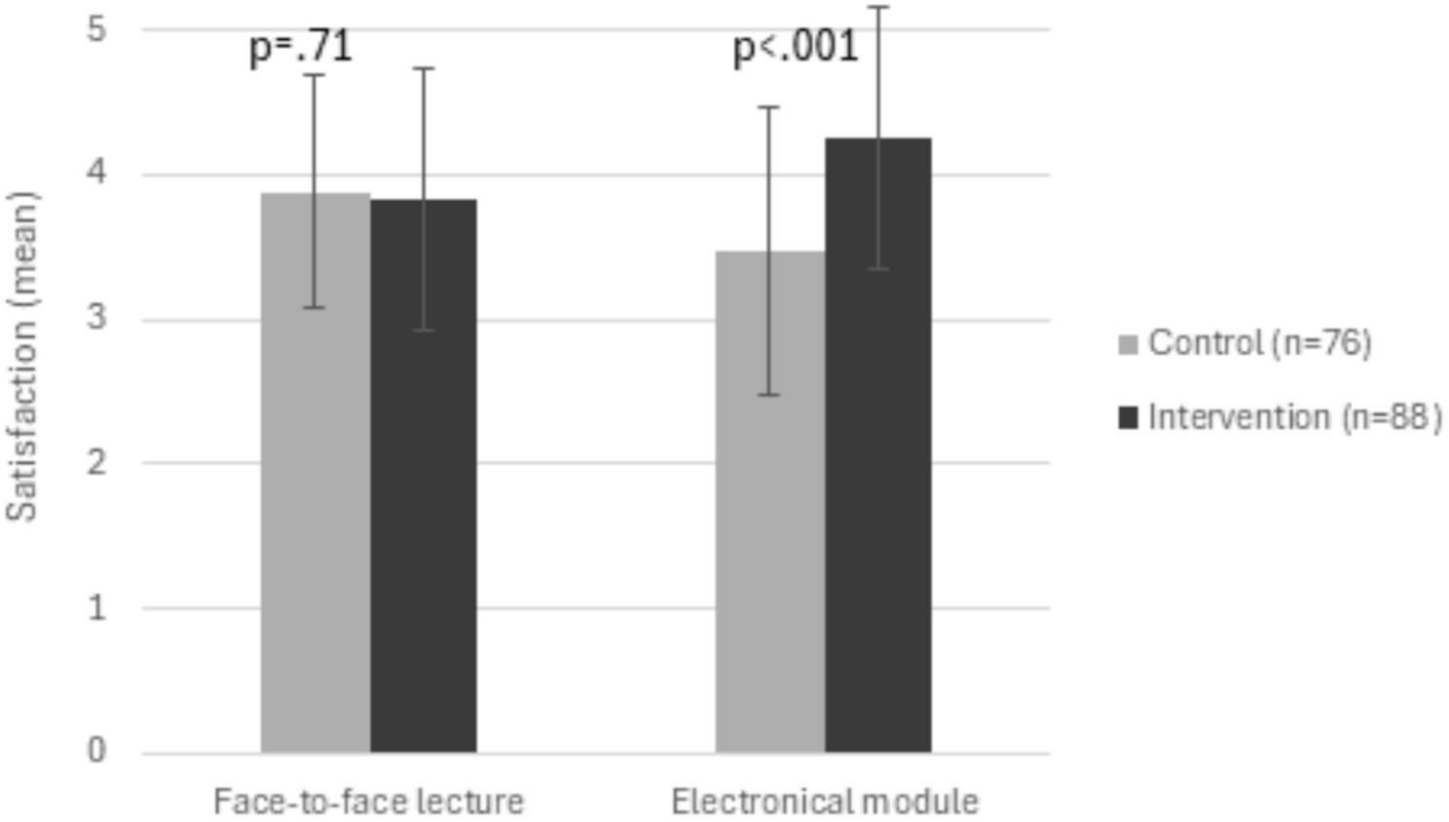
Student’s mean satisfaction with the course **Figure 3** shows students’ mean satisfaction scores (range: from 0–5) with the face-to-face lecture and the electronical module (i.e. the online learning or knowledge assessment only in the intervention group and control group, respectively)

The intervention group reported more overall engagement with the teaching contents compared to the control group (4.3 ± 0.9 versus 4.0 ± 0.8, difference 0.29, 95%CI 0.03 to 0.54, *p* = 0.03) as well as higher satisfaction with the electronic contents (4.3 ± 0.9 versus 3.5 ± 1, difference 0.78, 95%CI 0.48 to 1.07, *p* < 0.001). Students’ satisfaction with the face-to-face lecture was comparable between groups (3.8 ± 0.9 versus 3.9 ± 0.8, difference − 0.05, 95%CI -0.33 to 0.22, *p* = 0.71) (Fig. 3, Appendix 2).

### Qualitative analysis

#### Students’ free-text responses to written case vignettes

**Fig. 4:**
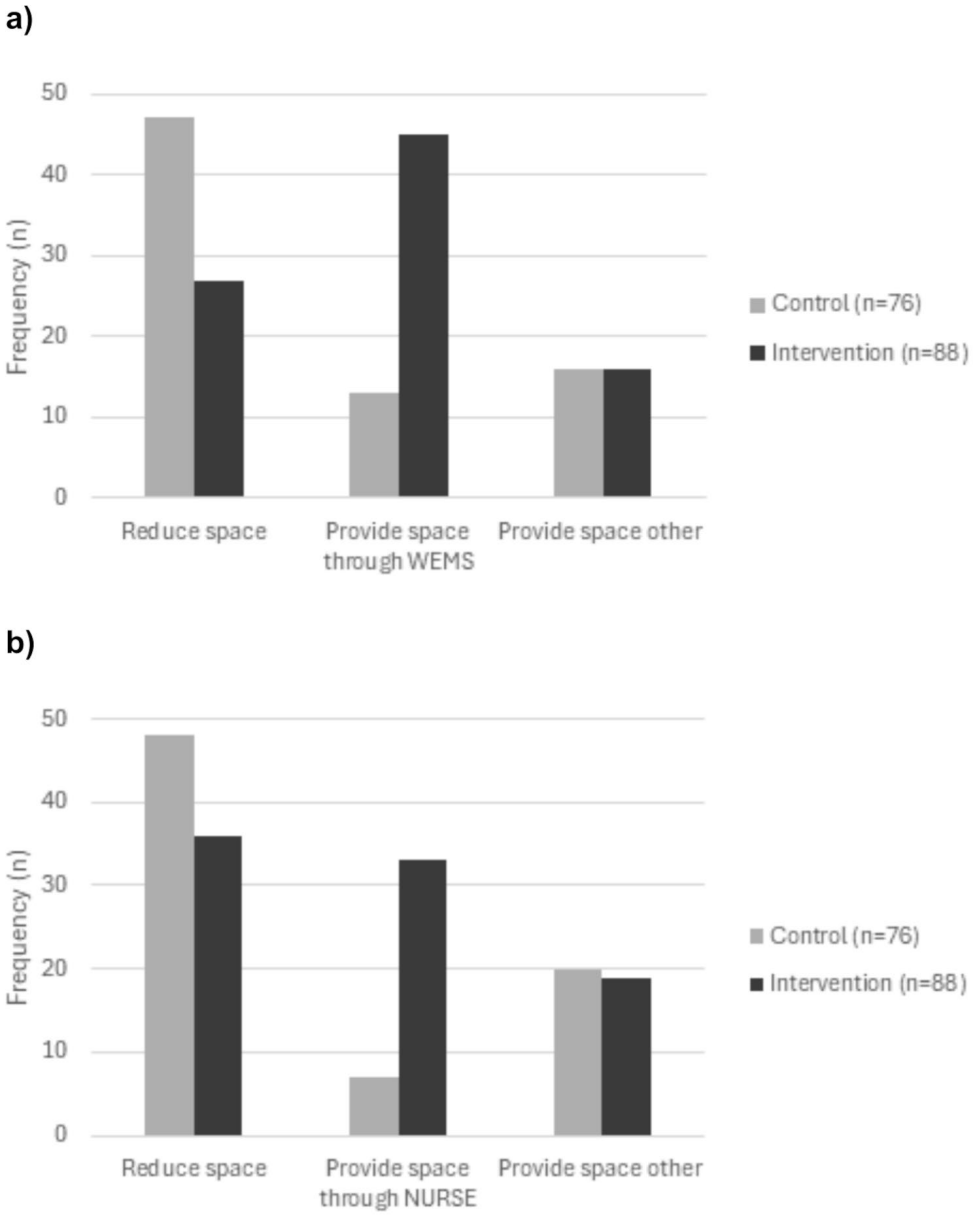
(**a**) Students’ reactions to a patient expressing concern **Figure 4a** shows in what frequency students reacted to a written case vignette of a patient expressing concern. Students’ answers were categorized as either reducing conversational space, providing space, or providing space by addressing the patient’s concern using the WEMS communication technique Abbreviations: WEMS, Waiting, Echoing, Mirroring, Summarizing (**b**) Students’ reactions to a patient expressing anger **Figure 4b** shows in what frequency students reacted to a written case vignette of a patient expressing anger. Students’ answers were categorized as either reducing conversational space, providing space, or providing space by addressing the patient’s emotion using the NURSE communication technique Abbreviations: NURSE, Naming, Understanding, Respecting, Supporting, Exploring

Examples of students’ free-text responses to two written case vignettes of patients expressing concern (WEMS-exercise) and anger (NURSE-exercise) are presented in Table 1. In both case vignettes, students in the intervention group more frequently responded using patient-centered communication techniques (i.e., WEMS or NURSE techniques) compared to the control group (Fig. 4a and b; Table 2). For example, a student allocated to the intervention group responded to the concerned patient by using the patient-centered Mirroring-technique (WE**M**S): *“It seems to me that you’re concerned that something isn’t right?“.* Also, students in the intervention group less frequently provided space-reducing responses compared to the control group (Fig. 4a and b; Table 2). For example, a student allocated to the control group responded to the upset patient using a space-reducing, focused question: *“Thank you for your patience! I’m here for you now. How is your health condition today?“.* Overall, qualitative analysis suggests that the intervention group performed better at transferring theory of patient-centered communication techniques to patient case vignettes.

#### Students’ qualitative feedback on the course

The written feedback on the online learning intervention was mostly positive. Students highlighted the animated explanatory videos and interactive films, which were perceived appealing and instructive. In addition, students expressed the desire to have more theory content presented in such online learning formats and being able to choose learning times independently. Moreover, it was desired to use face-to-face lessons mainly for practicing. Redundancies of lecture and online learning as well as somewhat unclear instructions to the video annotation task (see Appendix 1) were negatively emphasized.

## Discussion

This randomized, controlled trial assessed the impact of a new blended learning course on medical students’ communication skills, yielding three main results. First, in a knowledge assessment including both theory and practical questions, students in the intervention group performed significantly better with a difference of approximately 17% points compared to the control group. Second, students in the intervention group reported higher overall satisfaction with the course compared to students in the control group. Third, qualitative analysis of students’ free-text responses to patient cases vignettes with opportunities for empathic responses revealed that students in the intervention group more frequently responded using patient-centered communication techniques, while students in the control group more frequently provided space-reducing responses.

These results require further discussion to derive practical implications. First, evaluation methods of existing studies on blended learning have largely relied on a single assessment method, mostly on self-assessment tools to evaluate outcomes, typically focusing on students’ satisfaction or perceived improvements through questionnaires [2, 11, 15]. While self-assessment can provide important insights into subjective experiences, it has inherent limitations, such as susceptibility to bias and the inability to objectively measure actual skill acquisition or performance improvement. The present study addresses this gap by employing a multimethod evaluation approach, incorporating not only self-reported satisfaction but also objective measures, such as a knowledge assessment and video annotation tasks, to evaluate the impact of the intervention on students’ communication skills. This comprehensive evaluation strategy allows us to demonstrate that a blended learning format combining face-to-face teaching and online learning effectively enhances students’ understanding and application of communication techniques moving beyond reliance on subjective metrics. By integrating diverse assessment methods, this study contributes to a more nuanced understanding of the efficacy of blended learning in medical education. Until now, randomized, controlled trials attempting to measure the effects of blended learning in the context of medical education are inconclusive [27]. While positive effects are observed when compared to no intervention, the comparison with traditional learning formats yields mixed results. Some meta-analyses show little or no benefit from blended learning whilst others reported significantly better knowledge outcomes for blended learning [13, 27–29]. One randomized, controlled trial reported improved students’ Objective Structured Clinical Examination (OSCE) performance across various communicative domains including structuring communication and addressing emotions [9]. In line with these findings, the results of the present trial showed improvement in addressing emotions but not in communication structure. Communication structure, assessed through the video annotation task, may have been affected by ambiguity in the task instructions. Students’ qualitative feedback referred to the lack of clarity (e.g. *“I didn’t understand the task and couldn’t adjust the markings in the video.”*, see also Appendix 1). This unclear task might bias the quantitative results by lowering internal validity and thereby contributing to the lack of observed improvement.

Second, particularly since the COVID pandemic, literature has focused on possible benefits of blended learning from a students’ perspective [14, 30]. Pre-pandemic literature favors blended learning, or considers it equal to face-to-face learning, whereas some post-pandemic evidence suggests a preference for face-to-face teaching, possibly due to a desire for normalcy and social interaction [10, 14, 30]. Still, although a 2022 review calls for a thorough evaluation of the many new blended learning formats that have quickly emerged during the COVID pandemic, post-pandemic randomized, controlled trials are still scarce [31]. With social interaction no longer restricted, the present trial’s data suggest higher students’ satisfaction with blended learning compared to face-to-face teaching. Qualitative feedback indicated a preference for using online learning over face-to-face teaching for theoretical content and reserving face-to-face lessons primarily for practical exercises and hands-on practice. To optimize learning, theoretical content may be delivered via online learning platforms, allowing flexibility and self-paced learning, while face-to-face teaching should emphasize practical, interactive activities such as role-playing and simulations. Clear task instructions and regular student feedback are essential to ensure engagement and usability. This approach combines the strengths of both methods, enhancing medical communication training effectively and efficiently.

Third, the present results contribute to a series of heterogeneous findings on the effect of blended learning. A possible explanation for this heterogeneity may lie in the methodological diversity of blended learning formats [11, 27, 28]. Interestingly, previous research suggests that video-based online learning is superior to text-based online learning in terms of teaching certain practical clinical skills [32]. This aligns with a randomized, controlled trial from 2015 investigating the effect of a blended learning course on 124 medical and pharmacy students’ performance within objective structured clinical examination (OSCE) scores [9]. The course consisted of a video-based online learning module and group sessions for practicing. Students’ performance in all communication skill domains increased significantly. Moreover, a 2018 meta-analysis studying the effect of video-based “flipped classroom” format – a specific blended learning approach in which online learning is used explicitly as preparation before the face-to-face lesson. Results were in favor of flipped classrooms using pre-recorded videos before face-to-face class over traditional classrooms for health professions education [33]. The effectiveness of video-based learning is in line with the present results despite not having used flipped classroom. In particular, we found that interactive video scenarios were both, effective and preferred by students for teaching communication techniques. Namely, students in the intervention group more frequently provided empathic free-text responses by using patient-centered communication techniques compared to the control group after completing a video-based training program using an interactive video scenario. In these scenarios, students observed a physician-patient conversation, decided how the conversation should proceed and then viewed continuation. Notably, interactive video scenarios were mentioned as very useful in the students’ qualitative feedback. This aligns with previous literature demonstrating the effectiveness of interactive video scenarios in medical education [34]. On the other hand, the use of gamification elements within online learning formats has previously been shown to significantly enhance students’ motivation and encourage active participation [24, 25]. Despite not being mentioned by students within qualitative analysis, the implemented leaderboards might have contributed to the present results. In conclusion, the present findings hold important implications for the development of future blended learning formats in medical education: Online learning might be most effective, when applied video-based, interactive, and within blended learning format. These promising methods need to be further investigated for the optimal design.

This trial has limitations. First, the knowledge assessment used has not yet been validated, as it was designed specifically to comply with the learning objectives of the University of Basel medical curriculum which may impact reliability and validity [17]. Second, effects of the intervention for communication skills in real life daily practice were not assessed through OSCE or workplace-based assessment within this study. This may affect validity, for instance, as the knowledge assessment may not have fully captured all comprehensive aspects of communication. Future research should prioritize validating such tools to enhance measurement accuracy and generalizability. Third, 10 students in the control arm did not complete the knowledge assessment and were excluded from the analysis possibly introducing a risk of selection bias, as the excluded students may have had differing levels of prior knowledge or engagement compared to those who did complete the assessment.

## Conclusions

In conclusion, the use of a blended learning approach compared to a solely face-to-face course on medical communication, improved students’ communicative performance and satisfaction. As blended learning becomes increasingly important, further investigation into the optimal design for broad implementation in medical curricula is warranted. Specifically, combining video-based learning for theoretical instruction with practice-oriented face-to-face teaching holds promise for achieving these goals.

## Electronic supplementary material

Below is the link to the electronic supplementary material.

Supplementary Material 1

## Data Availability

Data is provided within the manuscript or supplementary information files.
