# Supplementary material for: Effectiveness of blended learning to improve medical students’ communication skills: a randomized, controlled trial
Source: BMC Med Educ. 2025 Mar 14;25:383. doi: 10.1186/s12909-025-06938-w (PMC11907784; doi:10.1186/s12909-025-06938-w)
Supplement: Supplementary file 1 — Supplementary Material 1 [file 12909_2025_6938_MOESM1_ESM.docx]

**Appendix**

**Appendix 1. Online learning contents**

| **Domain** | **Chapter** | **Content overview** | **Medium** | **Intervention** | **Control** |
| --- | --- | --- | --- | --- | --- |
| 1. Introduction | 1.1 Introduction video | The professor welcomes the students and gives information about randomization arms. | Talking-head Video | **✓** | **✓** |
| 2. Basics of medical communication | 2.1 Theory | Things to consider when initiating conversation with patients:   1. Confidential setting 2. Self-introduction 3. Body language 4. Conversation structure | Animated explanatory video | **✓** | **✗** |
|  | 2.2 Knowledge assessment | You (Dr. Schneider) enter the room of your new patient, Mrs. Müller, for the ward round. How do you address your patient? (SC, 2 points)   - Good day, my name is Dr. Schneider and I am here for the rounds. - Good day Mrs. Müller, I am your attending physician and I am here for the rounds. - Good day Mrs. Müller, my name is Dr. Schneider and I am your attending physician. I am here for the rounds. ✓ - Good day Mrs. Müller, my name is Dr. Schneider and I am here for the rounds. - Good day, my name is Dr. Schneider and I am your attending physician. I am here for the rounds.   Which statement regarding body language is correct? (SC, 2 points)   - Verbal communication is more important than non-verbal communication. - Studies show that the ideal physical distance to patients is around one meter. - Studies show that standing next to patients is perceived as more competent than sitting. - The physical distance to patients should be individually adjusted. ✓ - One should only sit next to patients if there is enough time.   How can a patient conversation be structured? (MC, 5 points)   - Communicating the available time frame ✓ - Book metaphor ✓ - NURSE communication techniques - WEMS communication techniques - Clarifying the conversation objectives at the beginning ✓   Which of the following statements are correct? (MC, 4 points)   - When delivering bad news, it is particularly important to create a confidential atmosphere and avoid possible interruptions. ✓ - Verbal communication is generally more important than non-verbal communication. - By sitting next to your patient on a chair, you signal that you are taking your time. ✓ - By sitting next to your patient on a chair, you signal that you are speaking on the same level as them. ✓   Should the introduction of medical personnel be the first part of a medical conversation? (K-Prim, 2 points)   - Yes, it builds trust and clarifies the role of the speaker. ✓ - No, the introduction takes up unnecessary time. - Yes, if the patient is new to the practice or clinic. - Not important in inpatient care as many people are involved in the treatment.   Which statements are correct? (K-Prim, 2 points)   - If patients are emotionally upset, one should interrupt their own conversation structure, if possible, to ask about their well-being. ✓ - One should consider the conversation objectives before the patient consultation. ✓ - One should consider the appropriate location before the patient consultation. ✓ - To adhere to the available conversation time, it can be helpful to announce the available time frame at the beginning. ✓   Scoring  Points total: 17  $score= \frac{points achieved\times100}{17}$ | Questionnaire / form | **✓** | **✓** |
| 3. The concept of physician- and patient-centered communication | 3.1 Theory | Two communication techniques in medical communication and when they are used:   1. Patient-centered communication  - Generating hypotheses - Open-ended questions  1. Physician-centered communication  - Clarifying hypotheses - Closed-ended questions - Focused questions | Animated explanatory video | **✓** | **✗** |
|  | 3.2 Knowledge assessment | Which strategy is not part of patient-centered communication? (SC, 2 points)   - Giving summaries to check understanding - The question: "Do you have chest pain?" ✓ - Showing empathy and understanding - The question: "What brings you here today?"   Which are attributes of physician-centered communication? (MC, 2 points)   - Closed-ended questions ✓ - Is hypothesis-generating - Is hypothesis-clarifying ✓ - Open-ended questions - Focused questions ✓   Which responses to the following conversation sequence are appropriate? (K-Prim, 2 points)  Physician: "What brings you here today?"  Patient: "It's just all a bit too much right now..."   - Respond with physician-centered communication - Respond with patient-centered communication ✓ - "A bit too much?" ✓ - "Do you have any physical symptoms?"   Example:  Physician: "Did you have any other symptoms besides nausea?"  Patient: "Yes, a pressure in the chest."  Which of the following statements are correct, which are incorrect? (K-Prim, 2 points)   - The physician is using a patient-centered communication strategy - The physician is asking an open-ended question - The physician is asking a focused question ✓ - The physician already has a hypothesis about what might be causing the symptoms. ✓   Which of the following statements are correct, which are incorrect? (K-Prim, 2 points)   - Patient-centered communication is important because it can help to capture less obvious symptoms. ✓ - Summarizing is only useful if the conversation was complex. Summarizing is unnecessary if there are no uncertainties. - The flow of conversation should be as smooth as possible. Longer interruptions should be avoided in a patient conversation. - Patients will have the impression that you have lost the thread due to too long pauses.   Which of the following statements are correct, which are incorrect? (K-Prim, 2 points)   - Patient-centered communication is more empathetic than physician-centered communication and should therefore be preferred whenever possible. - In the past, physician-centered communication was used. This technique has mainly been replaced by patient-centered communication. - Patients should never be interrupted during the conversation. - Patient-centered communication is becoming increasingly important as patients are more often psychologically burdened.   Scoring  Points total: 12  $score= \frac{points achieved\times100}{12}$ | Questionnaire / form | **✓** | **✓** |
| 4. WEMS communication techniques | 4.1 Theory | Patient-centered communication strategy to facilitate conversation consisting of the acronym:   - W – Waiting - E – Echoing - M – Mirroring - S – Summarizing | Animated explanatory video | **✓** | **✗** |
|  | 4.2 Training | Interactive film with multiple endings:   1. *Initial situation*: Patient Mrs. H. visits her general practitioner due to persistent symptoms after a flu infection. She describes her complaints and expresses her concerns. 2. *Interactive choice*: How should the physician respond? *Options*: Providing information, Waiting, Echoing, Mirroring, or Summarizing. 3. The conversation continues according to the chosen option. 4. Written feedback on whether the chosen technique was appropriate. 5. Encouragement to watch the other techniques as well (back to 2). | Live action film with actors | **✓** | **✗** |
|  | 4.3 Knowledge assessment | Patient Mr. M. visits his general practitioner and describes his concern:  Physician: *"Good day, Mr. M. What brings you to me today?"*  Patient*: "Good day, Physician. I would like to have a general check-up. Just to see if everything is okay."*  How would you respond here? (Free text answer, no points/ for qualitative evaluation only)  Which communication techniques are suitable in this situation? (MC)   - Providing medical information - Waiting ✓ - Echoing ✓ - Mirroring ✓ - Summarizing   Scoring  Points total: 5  $score= \frac{points achieved\times100}{5}$ | Questionnaire / form | **✓** | **✓** |
| 5. NURSE communication techniques for addressing emotions | 5.1 Theory | Patient-centered communication strategy for addressing emotions consisting of the acronym:   - N – Naming - U – Understanding - R – Respecting - S – Supporting - E – Exploring | Animated explanatory video | **✓** | **✗** |
|  | 5.2 Training | Interactive film with multiple endings:   1. *Initial situation*: Patient Mr. S. presents himself at the oncology consultation to discuss further therapy options after being diagnosed with lung cancer the previous week. During the conversation, the patient loses his composure and says he doesn't know how he will cope with the chemotherapy. 2. *Interactive choice*: How should the physician respond? *Options*: Providing information, Naming, Understanding, Respecting, Supporting, or Exploring). 3. The conversation continues according to the chosen option. 4. Feedback on whether the chosen technique was appropriate. 5. Encouragement to watch the other techniques as well (back to 2). | Live action film with actors | **✓** | **✗** |
|  | 5.3 Knowledge assessment | The resident visits her patient, Mrs. M., during rounds:  Physician: *"Good day, Mrs. M. I'm here for the ward round."*  Patient: *"It's about time. This hospital is an absolute dump!"*  How would you respond here? (Free text answer, no points/ for qualitative evaluation only)  Which communication techniques are suitable in this situation? (MC)   - Providing medical information - Naming **✓** - Understanding - Respecting - Supporting - Exploring **✓**   Scoring  Points total: 5  $score= \frac{points achieved\times100}{5}$ | Questionnaire / form | **✓** | **✓** |
| 6. Structuring medical information (book metaphor) | 6.1 Theory | Physician-centered communication technique to structure medical information. A conversation is structured similarly to a book:   1. Title 2. Table of contents 3. Chapter headings 4. Body text | Animated explanatory video | **✓** | **✗** |
|  | 6.2. Knowledge assessment | Video annotation task: Students observe a physician conducting a discharge conversation with a patient and must identify and mark the abovementioned techniques of the book metaphor at the appropriate times with a mouse click.  Scoring  Points total: 5 (1 point for each correctly annotated book metaphor element: title, table of contents, first chapter, second chapter, third chapter)  $score= \frac{points achieved\times100}{5}$ | Live action film with actors | **✓** | **✓** |

*Abbreviations: SC, Single-choice question; MC, Multiple-choice question; K-Prim, Keyed primary question type with four statements, each evaluated individually as true or false.*

**Appendix 2. Association of the online learning intervention with baseline characteristics and outcomes**

|  |  | **n** | **All** | **Control** | **Intervention** | **p-value** | **OR or difference (95%CI)** | **p-value** | **Adjusted OR or difference (95%CI)*** | **p-value** |
| --- | --- | --- | --- | --- | --- | --- | --- | --- | --- | --- |
| ***N*** |  |  | 164 | 76 | 88 |  |  |  |  |  |
| **Baseline Characteristics** |  |  |  |  |  |  |  |  |  |  |
| Female sex, n (%) |  | 164 | 105 (64.0%) | 48 (63%) | 57 (65%) | 0.83 | 1.07 (0.57, 2.03) | 0.83 | 1.1 (0.58, 2.09) | 0.771 |
| Age, mean (SD) |  | 164 | 22.0 (1.9) | 21.8 (1.9) | 22.1 (1.8) | 0.25 | 0.34 (-0.23, 0.91) | 0.246 | 0.34 (-0.23, 0.91) | 0.241 |
| **Primary Outcome** |  |  |  |  |  |  |  |  |  |  |
| Students' overall knowledge (from 0-100), mean (SD) |  | 164 | 65.8 (15.5) | 56.7 (15.3) | 73.6 (10.7) | **<0.001** | 16.97 (12.94, 21.01) | **<0.001** | 17.02 (12.95, 21.1) | **<0.001** |
| **Secondary Outcomes** |  |  |  |  |  |  |  |  |  |  |
| Students' knowledge on communication basics (from 0-100), mean (SD) | | 164 | 71.6 (22.4) | 61.4 (21.3) | 80.4 (19.6) | **<0.001** | 19.06 (12.76, 25.37) | **<0.001** | 19.26 (12.93, 25.59) | **<0.001** |
| Students' knowledge on patient- vs. physician-centered communication techniques (from 0-100), mean (SD) | | 164 | 74.1 (20.8) | 65.3 (23.1) | 81.7 (14.9) | **<0.001** | 16.39 (10.47, 22.31) | **<0.001** | 16.64 (10.71, 22.56) | **<0.001** |
| Students' knowledge on communication structure (from 0-100), mean (SD) | | 164 | 58.0 (35.1) | 54.2 (39.7) | 61.4 (30.4) | 0.19 | 7.15 (-3.68, 17.99) | 0.194 | 6.92 (-3.85, 17.68) | 0.206 |
| Students' knowledge on WEMS communicatuon techniques (from 0-100), mean (SD) | | 164 | 62.4 (24.1) | 52.4 (24.9) | 71.1 (19.9) | **<0.001** | 18.77 (11.87, 25.67) | **<0.001** | 18.85 (11.92, 25.79) | **<0.001** |
| Students' knowledge on NURSE communication techniques' (from 0-100), mean (SD) | | 164 | 62.6 (23.7) | 50.0 (21.1) | 73.5 (20.3) | **<0.001** | 23.48 (17.09, 29.88) | **<0.001** | 23.46 (17, 29.92) | **<0.001** |
| Students' responses to WEMS case vignette, n (%) | Reduce space | 164 | 74 (45.1%) | 47 (62%) | 27 (31%) | **<0.001** | 0.27 (0.14, 0.52) | **<0.001** | 0.27 (0.14, 0.53) | **<0.001** |
|  | Patient-centered techniques | 164 | 58 (35.4%) | 13 (17%) | 45 (51%) | **<0.001** | 5.07 (2.45, 10.51) | **<0.001** | 4.96 (2.38, 10.33) | **<0.001** |
|  | Provide space | 164 | 32 (19.5%) | 16 (21%) | 16 (18%) | 0.64 | 0.83 (0.38, 1.81) | 0.644 | 0.88 (0.4, 1.93) | 0.745 |
| Students' responses to NURSE case vignette, n (%) | Reduce space | 164 | 84 (51.2%) | 48 (63%) | 36 (41%) | **0.004** | 0.4 (0.21, 0.76) | **0.005** | 0.41 (0.22, 0.78) | **0.006** |
|  | Patient-centered techniques | 164 | 40 (24.4%) | 7 (9%) | 33 (38%) | **<0.001** | 5.91 (2.43, 14.39) | **<0.001** | 5.77 (2.36, 14.12) | **<0.001** |
|  | Provide space | 164 | 40 (24.4%) | 21 (28%) | 19 (22%) | 0.37 | 0.72 (0.35, 1.47) | 0.37 | 0.73 (0.35, 1.49) | 0.381 |
| **Students' Feedback** |  |  |  |  |  |  |  |  |  |  |
| Students' subjective engagement with learning content (from 1-5), mean (SD) | | 164 | 4.1 (0.8) | 4.0 (0.8) | 4.3 (0.9) | **0.03** | 0.29 (0.03, 0.54) | **0.03** | n.a.** | n.a.** |
| Students' satisfaction with face-to-face lecture (from 1-5), mean (SD) | | 164 | 3.9 (0.9) | 3.9 (0.8) | 3.8 (0.9) | 0.71 | -0.05 (-0.33, 0.22) | 0.707 | n.a.** | n.a.** |
| Students' satisfaction with online learning (from 1-5), mean (SD) | | 164 | 3.9 (1.0) | 3.5 (1.0) | 4.3 (0.9) | **<0.001** | 0.78 (0.48, 1.07) | **<0.001** | n.a.** | n.a.** |

**adjusted for age, sex.*

***not adjustable due to anonymous survey*

*Abbreviations: OR, odds ratio; CI, confidence interval; SD, standard deviation; n, number; WEMS, Waiting, Echoing, Mirroring, Summarizing; NURSE, Naming, Understanding, Respecting, Supporting, Exploring*
